# Aerobic exercise-induced lactate production: a novel opportunity for remodeling the tumor microenvironment

**DOI:** 10.3389/fgene.2025.1620723

**Published:** 2025-07-09

**Authors:** Xin Zhou, Jinliang Jiang, Jingjing Liu, Qinglu Wang, Ying Luo, Liping Wu

**Affiliations:** ^1^ Graduate School of Education, Shandong Sport University, Jinan, Shandong, China; ^2^ Department of Clinical laboratory, Zibo Central Hospital, Zibo, China

**Keywords:** lactylation, tumor microenvironment, Warburg effect, immune cells, aerobic exercise

## Abstract

Cancer, as one of the most deadly and burdensome diseases in the world today, causes irreversible damage to the body. However, due to the ineffective suppression of the inflammatory environment within tumors, identifying new therapeutic targets for cancer treatment has become an urgent issue. Recent studies have shown that lactate, a key product of glycolysis and exercise, is closely related to tumor development. Under normal conditions, lactate regulates various biological functions and can influence immune suppression, thereby interfering with tumor progression. Due to the Warburg effect, lactate levels in the tumor microenvironment (TME) are maintained at high levels. High levels of lactate can further induce the activation of an emerging post-translational modification—lactylation, which enhances the expression of relevant signaling pathways, including JAK-STAT and PI3K/Akt/mTOR. This, in turn, suppresses the body’s immune surveillance function and drives tumor progression through epigenetic-metabolic interactions. At the same time, aerobic exercise, as an important intervention for cancer, can enhance anti-inflammatory capacity by secreting muscle factors such as iris protein and tumor suppressor M, and it can also increase the infiltration of immune cells, including CD8^+^ T cells, improving tumor-killing abilities. Based on this, regular aerobic exercise can accelerate the clearance rate of lactate in the body, enhance lactate metabolism, reduce lactate concentration in the TME, and alleviate immune suppression. Therefore, this review explores the mechanisms behind the abnormal elevation of lactate in tumor cells and the occurrence of lactylation, proposing that aerobic exercise can intervene in the tumor process by inhibiting lactylation. The aim is to explore the interaction between aerobic exercise-induced lactylation modification and the tumor microenvironment, identify new therapeutic targets for solid tumors, and provide new ideas for their treatment.

## Introduction

Lactate, as the end product of glycolysis, plays a crucial role in energy metabolism and is also involved in the regulation of the cellular environment and signal transduction, influencing the biological environment through multiple pathways (Rabinowitz and Enerback, 2020). In addition, recent studies have shown that lactate can modulate and improve inflammation levels by triggering and activating a series of cellular signaling factors (Cassim and Pouyssegur, 2019). Numerous studies have shown that there is an inseparable relationship between tumors, inflammation, and oxidative stress. For example, a variety of inflammatory factors, including IL-6 and ROS, along with the abnormal activation of immune cells, collectively create a pro-cancer “inflammatory microenvironment.” Therefore, reducing and regulating the level of inflammation within the tumor microenvironment has become one of the key strategies in cancer treatment. Moreover, the Warburg effect demonstrates that tumor cells tend to rely on glycolysis for energy production even under adequate oxygen supply, leading to a significant increase in lactate levels within the tumor microenvironment (Fendt, 2024). Thus, lactate may serve as a “metabolic-immune hub” within the tumor microenvironment by regulating inflammation levels.

Recent studies have shown that excessive accumulation of lactate can induce the activation of an emerging post-translational modification—lactylation, which acts as an “enabler” of tumor development. Lactylation, on one hand, can inhibit the activity of immune cells, including CD8^+^ T cells, and on the other hand, it can activate the expression of pro-cancer pathways such as JAK-STAT, further promoting tumor progression (Wang et al., 2023). Interestingly, exercise, as the primary process mediating lactate production, also demonstrates significant potential in inhibiting tumor proliferation. Regular aerobic exercise not only enhances mitochondrial function to reduce excessive accumulation of lactate in the body, but also boosts the body’s metabolic capacity to accelerate lactate clearance, thereby lowering lactate concentration in the tumor microenvironment, alleviating immune suppression, and intervening in tumor progression. In summary, this suggests that the tumor-suppressive effects of exercise may be closely associated with lactylation and involve processes of immune suppression. Therefore, this review will comprehensively explore the interplay between exercise-regulated lactylation dynamics and the tumor microenvironment, with particular focus on the mechanisms by which exercise-mediated lactate modulation alleviates tumor-induced immunosuppression. This may contribute to a deeper understanding of how exercise inhibits tumor progression and offer novel insights for targeted interventions in tumor development and progression.

## Lactylation

### Lactate production and transport

In the human body, lactate is produced from pyruvate, the end product of glycolysis. The pathway of lactate generation is influenced by the availability of oxygen. Under adequate oxygen supply, energy is primarily produced via the tricarboxylic acid (TCA) cycle. However, under hypoxic conditions, pyruvate is reduced to lactate by lactate dehydrogenase (LDH), allowing glycolysis to continue as the main energy-producing pathway. It is noteworthy that, in order to better meet the demands of rapid cell proliferation, tumor cells preferentially utilize the glycolytic metabolic pathway to produce large amounts of lactate for energy, even under conditions of sufficient oxygen supply. This phenomenon is known as the Warburg effect, which has been demonstrated in various tumor models, including glioblastoma, pancreatic cancer, and breast cancer (Warburg et al., 1927). This metabolic mode, producing large amounts of lactate, allows lactate to act as a crucial metabolic intermediary and signaling molecule, further influencing the tumor microenvironment (TME).

In terms of lactate transport, lactate crosses the cell membrane with the assistance of monocarboxylate transporters (MCTs), which are important components of the cell membrane (Brizel et al., 2001). Currently, four types of MCTs are known to transport lactate, with MCT1 and MCT4 serving as the primary pathways. These transporters can finely adjust the lactate concentration gradient on the cell membrane based on the lactate levels within the body. MCT1, due to its high affinity for lactate, serves as the primary source of lactate influx. In contrast, MCT4, induced by hypoxia-inducible factor 1α (HIF-1α), has a lower affinity for lactate. It not only functions as the primary source of lactate efflux but also serves as a prognostic marker for poor outcomes in various cancers, drawing significant attention in cancer therapy (Li et al., 2022). HIF-1α, as an oxygen-dependent transcriptional activator that promotes mammalian development and accelerates tumor progression, can enhance tumor invasion by altering metabolic pathways and inducing the production of pro-angiogenic factors (Liu et al., 2022; Chen et al., 2021). Furthermore, HIF-1α can accelerate glycolysis by upregulating MCTs and promoting lactate efflux. It enhances the production of pyruvate by stimulating hexokinase 2 and fructose-2,6-bisphosphate in glycolysis, while also inhibiting pyruvate mitochondrial metabolism and activity through the induction of pyruvate dehydrogenase kinase-1, thereby supporting tumor cell growth and invasion (Zhang et al., 2019; Sabari et al., 2017). Overall, overactivation of HIF-1α in tumor cells and its impact on lactate accumulation is one of the key factors regulating tumor cells’ adaptation to the hypoxic environment.

### Lactylation modification and epigenetics

Epigenetics refers to the study of molecular modifications that regulate gene activity without altering the DNA sequence, thereby influencing gene expression. At the same time, epigenetics serves as a “gene switch” and a “critical bridge” connecting proteins with gene expression, playing a role in the onset and progression of various diseases, including autoimmune diseases and cancer (Peixoto et al., 2020). The origin of cancer is primarily due to the impaired activity and regulation of a set of oncogenes or tumor suppressor genes, which is closely associated with genetic and epigenetic alterations in the control of cell division (Park et al., 2022). The main mechanisms of epigenetic regulation include DNA methylation, histone modifications, and non-coding RNAs (ncRNAs). Unlike the two mechanisms of DNA methylation and ncRNA regulation at the “sequence level” and “gene level,” histone modifications can directly alter the spatial conformation of DNA polymers at the “chromatin structural level,” thereby leading to changes in the key transcription factors’ interaction with core gene regulatory regions of DNA, further altering transcriptional activity. This makes histone modification an important epigenetic regulatory mechanism (Moore et al., 2013; Chen and Xue, 2016; Bannister and Kouzarides, 2011). Common histone modifications include acetylation, phosphorylation, and ubiquitination. In recent years, in addition to these well-known post-translational modifications, a new protein modification, “lactylation,” has emerged in the scientific spotlight (Chen et al., 2022). In 2019, a team led by Professor Yingming Zhao at the University of Chicago discovered a new post-translational modification on histones using high-performance liquid chromatography-tandem mass spectrometry (Yang et al., 2022). Up to now, lactylation modifications have been identified in different parts of animal models (heart, eyes) (Sabari et al., 2017). Beyond animal models, multiple lactylation sites on proteins have also been observed in parasites (Yin et al., 2023)、fungi (Zhang and Zhang, 2024) and plants (Zhu et al., 2023) further demonstrating the widespread presence of lactylation modifications.

In addition, lactylation has been found to be closely linked to epigenetic regulation. Studies have shown that in a mouse model of peripheral nerve injury, p300 acts as a “writer enzyme” to catalyze histone lactylation, including H3K18la and H4K12la, thereby promoting the transcription of associated genes. Furthermore, the inhibition of p300 expression reduces histone lactylation processes, as well as downstream gene expression and pain-related behaviors (Pan et al., 2022). Moreover, results from *Bacillus* Calmette-Guérin (BCG) vaccination experiments demonstrate that the expression of H3K18la histone lactylation can enhance the vaccine response, thereby modulating the inflammatory reaction. This modification rewires cellular metabolism and further regulates gene expression (Ziogas et al., 2025). Therefore, understanding the relationship between histone lactylation and epigenetic regulation offers a novel perspective for the targeted treatment of various diseases.

### Histone/non-histone lactylation

Histones, as unique compounds, play a crucial role in the occurrence and progression of various diseases. They not only form modified groups by covalently binding to specific amino acid residues but can also serve as cleaved groups through proteolytic cleavage. Additionally, lactate can act as a precursor to stimulate histone lactylation, further altering the lactylation levels of histone lysine residues (Kla). Moreover, the regulation of Kla links metabolic, epigenetic, and transcriptional processes, further regulating gene expression in the tumor microenvironment (TME) and supporting the maintenance of the immune microenvironment.

In the context of the association between histones and lactate, lactate, as an epigenetic regulator, can directly bind to the lysine lactylation sites on histones, thereby inducing the polarization of macrophages toward the M2 phenotype. Existing research shows that lactate mainly consists of three chiral isomers, DL-lactate, D-lactate, and L-lactate (Li W. et al., 2024). Among these, L-lactate is known to be a precursor in the histone Kla (lactylation) process and is likely catalyzed by the reaction with acetyl coenzyme A. Subsequently, L-lactate is converted into acetyl coenzyme A, which, under specific enzymatic reactions, transfers the acetyl group to the lysine residues on histones, ultimately leading to histone lactylation. Extensive research indicates that histone lactylation preferentially affects enzymes involved in essential metabolic pathways such as lipids, amino acids, nucleotides, and others (Moreno-Yruela et al., 2022). Correspondingly, studies have also found that the processes of histone lactylation and delactylation can be regulated by the epigenetic activities of “writer,” “reader,” and “eraser” enzymes (Gaffney et al., 2020). Specifically, lysine acetyltransferase p300, a multi-substrate histone acetyltransferase, acts as a “writer” enzyme in the histone lactylation process. Additionally, p300 and its homolog CREB-binding protein (CBP) can promote lactylation modifications of High-Mobility Group Box-1 (HMGB1) in macrophages, suggesting that p300/CBP may play a key regulatory role in histone lactylation (Yang et al., 2024). On the other hand, Zn2+ and NAD + dependent histone deacetylases (HDAC1-3) may act as “eraser” enzymes involved in the dynamic regulation of histone delactylation, especially since HDAC1 and HDAC3 exhibit high delactylase activity in cells (Li X. et al., 2024).

In contrast to histone lactylation, non-histone lactylation primarily targets enzymes, transcription factors, and DNA damage repair proteins. It participates in dynamic cellular processes, including signal transduction and metabolic reprogramming, by altering enzyme activity and protein stability (Li et al., 2025). Compared to histone lactylation, non-histone lactylation functions more like an “epigenetic signal messenger.” Moreover, the lactylation of non-histone lysines is more challenging than that of histones. However, studies have indicated that lysines can undergo lactylation modification through a non-enzymatic reaction with lactylated glutathione (He et al., 2024). Moreover, the lactylation of non-histone proteins has been shown to be involved in tumor regulation. For instance, in hepatocellular carcinoma (HCC), Sirtuin3 (SIRT3) can intervene in the growth of liver cancer cells by regulating the lactylation of cyclin E2 (CCNE2) (Jin et al., 2023); In colorectal cancer (CRC), elevated lactate levels can increase lactylation of β-catenin, thereby inhibiting the activity of CRC cells and intervening in tumor progression (Miao et al., 2023). In summary, both histone and non-histone lactylation play roles in disease development through the regulation of gene expression, improvement of inflammatory responses, and other mechanisms, ultimately intervening in the tumor progression process.

## Lactylation and cancer

### Abnormal changes in lactate levels in tumor cells

**FIGURE 1:**
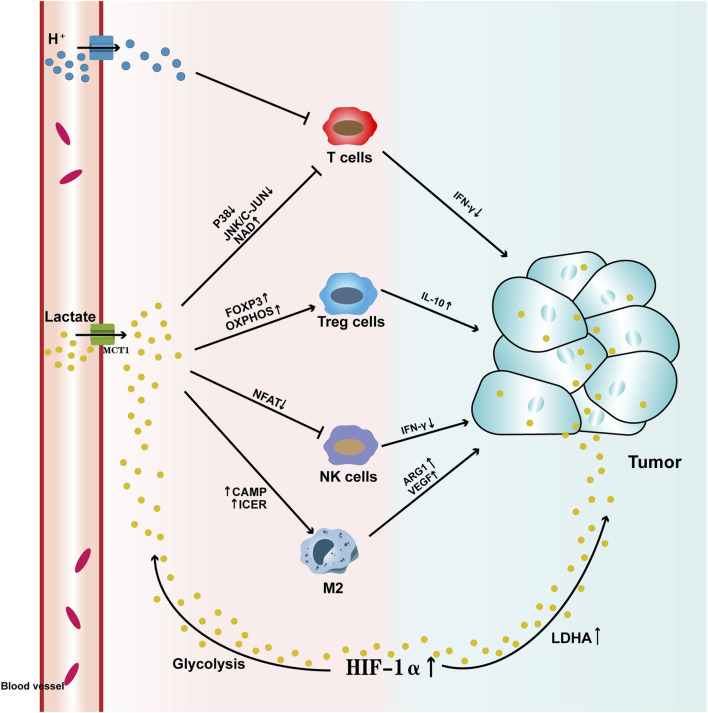
Pathways by which Lactate Affects Tumors Lactate enters the tumor microenvironment through the promotion of monocarboxylate transporter 1 (MCT1) across the cell membrane. High levels of lactate, on one hand, activate HIF-1α, which leads to abnormal expression of lactate dehydrogenase (LDHA), thereby increasing lactate levels within the tumor. At the same time, HIF-1α enhances glycolysis, increasing lactate production, which upregulates MCTs and facilitates lactate export. On the other hand, high lactate levels suppress the activity of T cells and NK cells, aberrantly activate Treg cells, and induce M2 macrophage polarization, which further elevates lactate levels in the tumor and promotes tumor progression. T cells: The high lactate tumor microenvironment not only accumulates a large amount of H+ ions, leading to substantial lactate accumulation, but also suppresses the activity of the p38 and JNK/c-JUN pathways. Additionally, elevated levels of nicotinamide adenine dinucleotide (NAD+) reduce T cell activity, leading to decreased IFN-γ levels and hindering T cell-mediated anti-tumor responses. Treg cells: The high lactate tumor microenvironment enhances the expression of the FOXP3 gene and accelerates oxidative phosphorylation (OXPHOS), which activates Treg cell activity, further increasing IL-10 levels and accelerating tumor progression. NK cells: The high lactate tumor microenvironment suppresses NFAT activation, thereby reducing NK cell activity and inhibiting IFN-γ production, which diminishes their tumor-killing ability. Macrophages: High lactate levels activate cAMP and ICER expression, inducing M2 macrophage polarization, upregulating the expression of ARG1 and VEGF, and accelerating tumor progression.

The elevated lactate levels in tumor cells are not only closely related to the Warburg effect but also contribute to the abnormal expression of various glycolytic enzymes and monocarboxylate transporters, such as lactate dehydrogenase A (LDHA), MCT1, and MCT4, through the upregulation and activation of HIF-1α and c-Myc. This results in the substantial accumulation of lactate in tumor cells (Chen et al., 2023). Compared to normal tissues, the serum lactate levels in tumor patients can reach 10–30 mM, while the lactate concentration in the tumor core can reach 50 mm (Ju et al., 2024) At the same time, the increase in lactate levels within tumor cells activates and accelerates lactylation processes, further aggravating tumor malignancy. For example, in bladder cancer, H3K18 lactylation promotes tumorigenesis by enhancing the expression of the oncogene Lipocalin-2 (LCN2) (Xie et al., 2023); in endometrial cancer, histone lactylation upregulates USP39 expression, which further interacts with PGK1 to activate the PI3K/AKT/HIF-1α signaling pathway, accelerating tumor progression (Wei S. et al., 2024); in colorectal cancer, the activation of G-protein coupled receptor 37 (GPR37) promotes the expression of LDHA by activating the Hippo pathway, thereby intervening in tumor progression (Zhou et al., 2023). In conclusion, the proliferation and growth of tumor cells are closely related to lactylation modifications (Figure 1).

### Lactylation and the tumor microenvironment

The tumor microenvironment (TME) refers to the environment in which tumor cells survive, primarily composed of innate immune cells such as macrophages, neutrophils, myeloid-derived suppressor cells, dendritic cells, and natural killer (NK) cells, as well as adaptive immune cells like T lymphocytes and B lymphocytes. Additionally, it includes stromal fibroblasts, blood and lymphatic vessel networks, and the inflammatory factors and chemokines they produce. The TME is closely related to tumor initiation, progression, and metastasis (de Visser and Joyce, 2023). The tumor microenvironment can influence the development of tumors by stimulating their growth, dormancy, invasion, and metastasis, thereby affecting patient survival rates. Furthermore, the TME contains a wide variety of innate immune cells, including macrophages, NK cells, and regulatory T cells (Tregs), whose presence is closely linked to the progression of the tumor (Ma et al., 2025).

### Macrophages

Macrophages are one of the important innate immune effector cells, originating from monocytes. They often adopt M1 and M2 phenotypes when infiltrating tumor tissues (Pan et al., 2020). These two phenotypes have opposing functions in targeting tumor cells. M1 macrophages primarily target tumor cells through the release of cytokines (such as TNF-α) and phagocytosis, playing a role in promoting inflammation. In contrast, M2 macrophages suppress inflammation by releasing cytokines, promote immune suppression (such as IL-10, IL-13), facilitate tumor cell metastasis through factors like EGF and MMP, and also promote angiogenesis. Tumor-associated macrophages (TAMs) are macrophages recruited to the tumor microenvironment during tumor development, where they promote tumor cell metastasis and proliferation (Wynn et al., 2013). They undergo metabolic reprogramming based on changes in tumor lactate levels, shifting towards an immune-suppressive phenotype similar to M2 macrophages (Viola et al., 2019). The increased lactate levels in the tumor microenvironment can further induce M2 macrophage polarization by affecting HIF-1α, ERK-STAT3 signaling pathways, and G-protein-coupled receptor 132 (GPR132) (Li et al., 2023). GPR132, which is highly expressed on the surface of macrophages, can activate cyclic AMP (cAMP) in response to extracellular lactate levels and, through the induction of the cAMP early repressor (ICER), further upregulate the expression of arginase 1 (ARG1), VEGF, and HIF-1α, playing a crucial role in the induction of the macrophage pro-inflammatory phenotype (Prenen and Mazzone, 2019). Similarly, in the TME, the level of histone lactylation can alter macrophage phenotype and function (Han et al., 2023). For example, when lactate levels are high, M2 macrophages exhibit higher vitality and better adaptability compared to M1 macrophages. Therefore, the lactate level within the tumor environment plays a key signaling role in inducing macrophage polarization (Liu et al., 2019) (Figure 1)

### Natural killer cells (NK cells)

Natural Killer (NK) cells are one of the innate immune cells with strong cytolytic activity. They participate in early tumor immune surveillance by releasing perforin, granzymes, and cytokines, thereby enhancing tumor-killing capacity (Liu et al., 2021). When lactate levels in the tumor microenvironment are excessively high, NK cell activity is significantly reduced. High lactate levels directly inhibit the cytolytic function of NK cells, and also indirectly suppress their activity by increasing the number of myeloid-derived suppressor cells (Terren et al., 2019). Furthermore, elevated lactate levels can inhibit the activation of the nuclear factor of activated T cells (NFAT) in NK cells, further suppressing the production of IFN-γ, which reduces their ability to recognize and kill tumor cells (Jin et al., 2025). Studies have shown that in a melanoma mouse model, the release of lytic granules, as well as the secretion of IFN-γ and TNF-α, is greatly reduced with the decrease in lactate levels in the tumor microenvironment, leading to a diminished cytotoxic response against tumor cells (Brand et al., 2016) (Figure 1)

### Regulatory T cells, Tregs

Tregs (regulatory T cells) are a type of suppressive CD4^+^ T cells primarily regulated by the expression of FOXP3. They inhibit the inflammatory response in the tumor microenvironment (TME) by suppressing effector functions and the migration of immune cells, helping the body maintain immune homeostasis. However, due to changes in the tumor environment, Tregs can shift to suppress anti-tumor immunity (Tay et al., 2023). In tumor microenvironments with high lactate levels, FOXP3 can further reprogram Treg cells by inhibiting c-Myc and glycolysis, and enhancing oxidative phosphorylation (OXPHOS), allowing Tregs to better adapt to the high-lactate environment (Ding et al., 2024). Meanwhile, Tregs produce suppressive cytokines such as IL-10, further inhibiting the activity of effector T cells and accelerating tumor cell invasion (Vilbois et al., 2024). Additionally, the lactate influx mediated by MCT1 is particularly important for maintaining the suppressive activity of Treg cells. In TME with high lactate levels, Treg cell activity and recruitment capabilities are significantly enhanced, further suppressing anti-tumor immunity (Kumagai et al., 2022). Overall, changes in lactate levels in the tumor microenvironment are closely linked to Treg cell activity (Figure 1).

### T lymphocyte, T cells

T cells are the main force of the immune system in killing tumor cells, primarily by recognizing tumor antigens and modulating the immune microenvironment to combat tumors (Kiri and Ryba, 2024; Liu et al., 2023). However, elevated lactate levels in the tumor microenvironment have a detrimental effect on T cell activity. T cell receptors can trigger the p38 and JNK/c-JUN pathways, which are highly associated with IFN-γ, to resist tumors. However, high lactate levels in the tumor microenvironment suppress the activity of both pathways and increase the levels of nicotinamide adenine dinucleotide (NAD^+^) to induce T cell apoptosis, thereby inhibiting their tumor-killing capability (Xia et al., 2017; Mendler et al., 2012). Additionally, due to the transmembrane lactate concentration gradient, the high-lactate tumor microenvironment accumulates a large amount of H^+^, promoting the accumulation of endogenous lactate, which further impairs T cell anti-tumor activity (Raychaudhuri et al., 2024). Therefore, in the TME, T cells must not only compete with tumor cells for glucose but also avoid the acidification of the cellular environment caused by MCT-mediated lactate transport (Figure 1).

### Lactylation and the PI3K/Akt/mTOR signaling pathway

The PI3K/Akt/mTOR signaling pathway is one of the most commonly dysregulated pathways in tumors, playing a crucial role in promoting tumor initiation, progression, and treatment. It consists of phosphoinositide 3-kinase (PI3K), protein kinase B (Akt), and the mechanistic target of rapamycin (mTOR), and is also a complex signaling axis composed of multiple upstream and downstream regulatory factors (Yu et al., 2022). The PI3K/Akt/mTOR pathway has been shown to be excessively activated in many solid tumors. For instance, in liver cancer, aberrant activation of the PI3K/PTEN/Akt/mTOR pathway upregulates matrix metalloproteinase 9 (MMP-9), contributing to tumor invasion and metastasis (Li et al., 2024c). Moreover, key components like PI3K kinase in this pathway have been found dysregulated frequently in various cancers (Prvanovic et al., 2021). Studies indicate that PI3K can influence glioblastoma development as a growth-promoting factor and intervene in the progression of hepatocellular carcinoma (HCC) by affecting related signaling pathways.

mTOR is one of the key regulatory factors in the PI3K/Akt/mTOR signaling pathway, consisting of the mechanistic target of rapamycin complex 1 (mTORC1) and the mechanistic target of rapamycin complex 2 (mTORC2). It influences the activity of the pathway through both positive and negative feedback mechanisms. mTORC1 and mTORC2 have distinct functions: mTORC1 regulates cell growth and metabolism by integrating signals from various growth factors and energy supply, while mTORC2 is responsible for regulating cell proliferation and survival (Song et al., 2019). Additionally, mTOR can influence tumor initiation and progression through post-translational modifications, such as phosphorylation. Studies show that upon activation of mTOR, the downstream S6K can phosphorylate insulin receptor substrate 1 (IRS-1), reducing the activity of PI3K and, through negative feedback, attenuating the activity of the PI3K/Akt/mTOR pathway, thereby inhibiting tumor cell activity (Leiphrakpam and Are, 2024).

Adenosine monophosphate-activated protein kinase (AMPK) is also an integrated metabolic sensor in the PI3K/Akt/mTOR signaling pathway, primarily regulating cell survival metabolism, cellular homeostasis, and maintaining energy balance (Herzig and Shaw, 2018). AMPK is closely associated with tumor development. On one hand, AMPK acts as a negative regulator of mTORC1. When activated, it reduces mTORC1’s inhibitory effects on autophagy-related proteins, inducing autophagy and apoptosis in tumor cells. On the other hand, AMPKα1, as the catalytic subunit of AMPK, inhibits various inflammatory transcription factors and reduces the secretion of pro-inflammatory cytokines such as tumor necrosis factor α (TNF-α), monocyte chemoattractant protein 1 (MCP-1), IL-1β, and interferon γ (IFN-γ), thereby interfering with tumor cell growth (Hsu et al., 2022). Additionally, there is a close relationship between lactate levels and AMPK activity. Studies have shown that in hepatocellular carcinoma, elevated lactate levels lead to AMPK inactivation, which further enhances the production of anti-ferroptotic monounsaturated fatty acids and accelerates tumor invasion. Blocking lactate uptake reactivates AMPK, promotes ferroptosis, and enhances anti-tumor activity (Zhao et al., 2020). Therefore, this review suggests that the accumulation of high levels of lactate in tumor cells may activate AMPK, which in turn reduces mTORC1 activity and indirectly weakens the PI3K/Akt/mTOR signaling pathway, thus intervening in tumor progression. However, since this pathway involves many proteins and signaling molecules, the specific molecular mechanisms are still unclear and require further investigation.

## Exercise and lactation

### Exercise and lactic acid

As a “multidimensional regulatory hub” against tumors, exercise has been shown to inhibit the occurrence and progression of various types of cancer through biological mechanisms such as enhancing immune system function, improving metabolic levels, and regulating inflammatory responses (Wernhart and Rassaf, 2025). During physical activity, the body’s temperature quickly rises, and a significant amount of cytokines such as catecholamines, myokines, and irisin are released. This release further activates the sympathetic nervous system, increases blood flow, and triggers a stress response affecting tumor metabolism and overall body homeostasis. After prolonged regular aerobic exercise, these stress responses can improve blood perfusion within the tumor, enhance immunogenicity, and consequently slow tumor progression. Research has shown that in rodent models, implementing aerobic exercise interventions, including voluntary activities like running on a wheel and swimming, significantly reduces the incidence of tumors, as well as their growth and metastasis (Chen X. et al., 2025). It is undeniable that tumors are not isolated entities separate from other body tissues; thus, their metabolism is inevitably influenced during exercise.

Lactate is not only an important metabolic product in the body, but also serves as a “dynamic buffering regulatory valve” between the exercise and immune networks. Different types of exercise influence the metabolic levels of lactate in the body. Research shows that high-intensity exercise can rapidly increase lactate levels by 5–10 mM through glycolysis, further promoting macrophage polarization from the M1 phenotype to the M2 phenotype (Chang and Tang, 2024). In contrast, regular moderate-intensity exercise induces a modest increase in lactate levels (2–4.5 mm), regulating lactate-metabolizing enzymes such as PDH and LDHA, balancing glycolysis and oxidative phosphorylation, and stabilizing HIF-1α to adapt to fluctuations in oxygen availability. This, in turn, enhances mitochondrial oxidative capacity, improves metabolic flexibility, and increases lactate clearance efficiency (Ren and Zhang, 2024; Chang et al., 2024). Therefore, exploring the “exercise-lactate-tumor” axis provides a different perspective for a better understanding of immune metabolism and epigenetics.

### Exercise interferes with tumor growth by altering lactate levels

Histone lysine lactylation modification primarily involves the addition of a lactyl group to lysine residues at various sites on histones, forming ε-N-lactyl lysine. This modification subsequently affects the interaction between histones and DNA, leading to chromatin alterations that interfere with the onset and progression of various diseases, including cancer, inflammatory diseases, cardiovascular diseases, and neurodegenerative disorders (Li et al., 2024d). In 2019, the team led by Zhao Yingming first identified 28 lactylation sites on core histones in mouse bone marrow-derived macrophages and human HeLa cells. Building on this, further research has focused on the lactylation profiling of lysine residues in various proteins at different sites, expanding the study of lactylation to encompass a wide range of major systemic diseases. For example, in inflammatory diseases, the activation of H3K14la lactylation upregulates the transcription of p53 in lung epithelial cells, inducing alveolar structural damage, inflammatory cell infiltration, and a decline in lung function in mice (Wang et al., 2025); In cardiovascular diseases, the activation of H3K14la and H3K9la lactylation leads to abnormal expression of the lumican protein, accelerating the progression of calcific aortic valve disease (Huang et al., 2024); In neurodegenerative diseases, H3K18la lactylation accelerates brain aging and the phenotype of Alzheimer’s disease by activating NF-κB signaling and upregulating the expression of IL-6 and IL-8 (Wang Y. et al., 2024). More importantly, excessive accumulation of lactate further induces histone lysine lactylation at various sites, which regulates gene expression and modulates the immune microenvironment homeostasis, thereby influencing tumor initiation and progression (Li et al., 2024e). Studies have shown that hypoxia can promote the process of H3K9la lactylation, enhancing the transcription of LAMC2 to facilitate the proliferation of esophageal squamous cell carcinoma (ESCC) (Zang et al., 2024); The acceleration of the H3K18la lactylation process enhances immune evasion in non-small cell lung cancer (NSCLC) by activating POM121, which induces the expression of PD-L1 (Zhang et al., 2024); The activation of H4K12la lactylation promotes drug resistance in ovarian cancer cells by upregulating SE-mediated aberrant RAD23A expression (Lu et al., 2025). Exercise can intervene in the lactylation process by regulating the levels of circulating and intracellular lactate. Studies have shown that exercise can reduce the overexpression of several glycolysis-related genes, including Aldoa, Pfkm, and Pkm, and decrease the expression of YTHDF2 in cardiomyocytes, thereby improving the lactylation process and preventing myocardial ischemia-reperfusion injury (Xu et al., 2024); Moreover, a multi-omics study on the effects of moderate-intensity exercise on protein lactylation in mouse muscle tissue showed that moderate-intensity exercise can alter lactate concentration by reducing the expression of Mtatp8, Atp5mg, and Atp5po, thereby downregulating lactylation levels and improving the expression of protein levels in mice (Chang et al., 2024). Therefore, this review will summarize the histone lactylation at three different sites closely related to tumor progression, unraveling the mystery of “exercise—lactylation—tumor.”

### H3K9la

**FIGURE 2:**
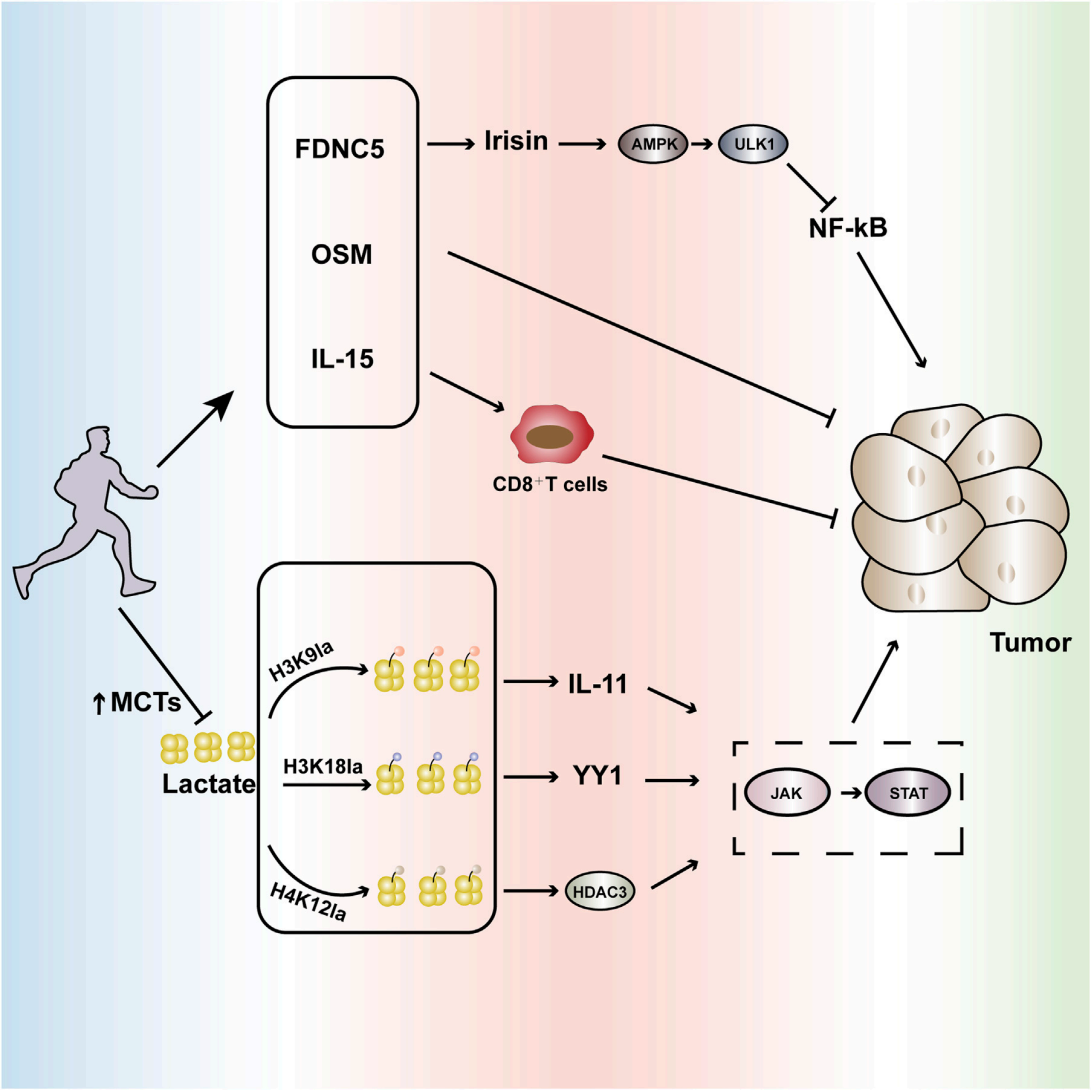
Aerobic exercise interferes with tumor progression. Regular aerobic exercise can slow tumor growth by affecting cytokines such as FNDC5, OSM, and IL-15. Additionally, it enhances the expression of monocarboxylate transporters (MCTs), reducing lactate levels and further influencing the lactylation process at abnormally expressed sites in tumors such as H3K9la, H3K18la, H4K12la, etc. This modulation inhibits the aberrantly activated JAK-STAT signaling pathway, intervening in tumor occurrence and development. FNDC5(Fibronectin Type III Domain-Containing Protein 5): Regular aerobic exercise can increase the secretion of FNDC5, stimulating the production of irisin. This, in turn, induces autophagy via the AMPK-ULK1 signaling pathway and inhibits NF-kB activation, influencing tumor development. OSM (Oncostatin M): Regular aerobic exercise promotes the secretion of OSM, further impacting tumor growth. IL-15: Aerobic exercise can modulate IL-15, enhancing the infiltration of CD8^+^T cells and improving their tumoricidal capabilities. H3K9la: High levels of lactate induce the lactylation of H3K9, leading to the overproduction of IL-11, which further activates the JAK-STAT pathway, accelerating tumor progression. H3K18la: High lactate levels induce the activation of lactylation at H3K18, leading to the overexpression of the transcription factor YY1, which accelerates tumor progression through the activation of the JAK-STAT pathway. H4K12la: High lactate levels induce the activation of lactylation at H4K12, enhancing the HDAC3 loop highly associated with tumors and increasing the activity of the JAK-STAT pathway, promoting tumor progression. Through these multifaceted biochemical pathways, regular aerobic exercise significantly influences the tumor microenvironment, potentially serving as a complementary strategy in cancer prevention and management.

H3K9la specifically refers to the lactylation modification of the ninth lysine on histone H3, which can drive gene expression reprogramming and assist tumor cells in evading immune surveillance. Taking head and neck squamous cell carcinoma (HNSCC) as an example, the tumor microenvironment in HNSCC patients is at a high lactate level, which further induces H3K9la lactylation. H3K9la lactylation promotes the secretion of IL-11, activating the JAK2-STAT3 signaling pathway, leading to CD8^+^ T cell exhaustion and dysfunction, thereby accelerating the occurrence and progression of HNSCC (Wang R. et al., 2024). Exercise can promote immune mobilization and restore the activity of CD8^+^ T cell infiltration into tumors. Studies have shown that regular moderate-intensity aerobic exercise can increase the number of CD8^+^ T cells in mice with breast cancer, enhance their trafficking ability and infiltration into tumors, thereby strengthening their anti-tumor capacity (Gomes-Santos et al., 2021); Furthermore, in a pancreatic ductal adenocarcinoma mouse model, it has also been shown that aerobic exercise can promote the infiltration of CD8^+^ T cells into the tumor microenvironment of both pancreatic ductal adenocarcinoma mice and patients, slowing down the tumor growth rate (Kurz et al., 2022). Therefore, this review suggests that aerobic exercise can reduce H3K9la lactylation, which accelerates tumor progression, by restoring the ability of CD8^+^ T cells to kill tumors (Figure 2).

### H3K18la

In addition to H3K9la lactylation, H3K18la has also been shown to be activated in tumor tissues. H3K18la is formed by the enzymatic attachment of lactate to a specific lysine residue on histone H3. This modification accelerates tumor proliferation and metastasis by enhancing the expression of oncogenes, including TTK and BUB1B, as well as regulating metabolic pathways. Taking bladder cancer (Bca) as an example, studies have found that the excessive elevation of lactate levels in the tumor microenvironment of Bca patients induces the activation of H3K18la. The key transcription factors driven by H3K18la, YBX1 and YY1, further enhance the resistance of Bca patients to cisplatin, which is unfavorable for tumor treatment (Li et al., 2024f). Meanwhile, studies have shown that YY1 can also slow down the transcriptional activation of IFN-γ by activating the JAK-STAT pathway and forming a protein complex with the nuclear factor AP2, thereby accelerating tumor invasion (Gordon et al., 2006). Regular aerobic exercise can enhance the number of CD8^+^ T cells by restoring mitochondrial metabolism, thereby promoting the production of IFN-γ. Studies have shown that moderate-intensity aerobic exercise can reduce the loss of mitochondrial activity in colorectal cancer mice, promote the infiltration of CD8^+^ T cells into the tumor microenvironment, increase IFN-γ production, and slow down tumor growth (Voltarelli et al., 2024); In terms of oxidative stress and inflammation, studies have shown that 24 weeks of aerobic exercise can increase the IFN-γ levels in blood samples of elderly individuals with cognitive frailty, thereby reducing inflammation (Ye et al., 2024). Therefore, this review suggests that aerobic exercise can promote the production of IFN-γ by improving the infiltration of immune cells into tumors, thus intervening in the H3K18la lactylation to accelerate the progression of tumor development (Figure 2).

### H4K12la

H4K12la is a lactylation modification located at lysine 12 of histone H4, playing a significant role in metabolically active diseases such as tumors. In studies of fibroblast collagen synthesis, high levels of lactate induced the activation of H4K12la lactylation. This, in turn, accelerated the binding of macrophages and the TGF-β gene promoter through the enhancement of the HDAC3 loop, thereby promoting collagen synthesis in fibroblasts (Zou et al., 2025). HDAC3 is a member of the class I HDAC family and is highly expressed in tumor tissues. It can not only accelerate tumor development by activating the JAK-STAT pathway, but also promote the abnormal activation of PD-L1 through binding with ZFP36L1, leading to T cell exhaustion and accelerating the progression of tumor developmen (Qiu et al., 2023; Wei X. et al., 2024). Aerobic exercise can improve the tumor microenvironment by regulating hypoxia, thereby reducing the abnormal activation of PD-L1. Studies have shown that, in melanoma mice, the combination of aerobic exercise and anti-PD-L1 treatment leads to significantly better tumor suppression effects compared to the control grou (Yan et al., 2023); Moreover, studies on a lung cancer mouse model have shown that aerobic exercise can significantly reduce the PD-L1 mRNA levels in lung cancer mice, further improving the expression levels of immune checkpoint proteins in the tumor tissue and intervening in tumor growth (Ge et al., 2022). Therefore, this review suggests that aerobic exercise can regulate the tumor microenvironment by reducing the abnormal activation of PD-L1, thereby improving tumor invasion caused by H4K12la lactylation (Figure 2).

As mentioned above, lactylation at the H3K9La, H3K18La, and H4K12La sites can accelerate tumor progression by activating the JAK-STAT pathway. The JAK-STAT signaling pathway is abnormally activated in various immune-mediated diseases, including melanoma, glioblastoma, as well as lung cancer, liver cancer, and breast cancer. Therefore, inhibiting the excessive activation of the JAK-STAT signaling pathway can effectively reduce tumor occurrence and progressio (Xue et al., 2023). Studies show that aerobic exercise can alleviate cardiac hypertrophy and inflammation by upregulating miR-574-3p and inhibiting the IL-6/JAK/STAT pathwa (Chen et al., 2024); Moreover, aerobic exercise can reduce the activity of the JAK-STAT pathway through the potential target NR1D1, thereby improving muscle function in mice (Yao et al., 2024). Therefore, regular aerobic exercise can reduce tumor lactate metabolism by enhancing the expression and function of MCTs, decrease lactylation modifications and the secretion of related cytokines and inflammatory responses, regulate the immune environment, and inhibit the abnormal activation of the JAK-STAT pathway to regulate tumor progression (Benitez-Munoz et al., 2024).

In addition to reducing the tumorigenic process through decreased lactylation modifications and cytokine-mediated pathways, exercise can also influence the tumor microenvironment by inducing myokines. Fibronectin type III domain-containing protein 5 (FNDC5) is a type I membrane protein that, upon cleavage, can form a new hormone called irisin, which participates in tumor cell proliferation and invasion (Cebulski et al., 2023). Studies have shown that exercise can stimulate the production of irisin by inducing FNDC5, which then mediates autophagy through the AMPK-ULK1 signaling pathway, inhibits NF-kB activation, and exerts anti-inflammatory effects that interfere with tumor progression (Li et al., 2018). Furthermore, exercise can stimulate the secretion of Oncostatin M (OSM), which suppresses tumor development. Research demonstrates that in a murine model of breast cancer, aerobic exercise intervention significantly increased serum OSM mRNA expression, which then upregulated caspase activity, inhibiting the proliferation of breast cancer cells (Kim et al., 2022). In addition, aerobic exercise can improve the instability of the endothelial barrier during tumorigenesis by regulating redox-sensitive GTPase-activating proteins such as Rho (Wolff et al., 2015). In conclusion, exercise, as a “multidimensional regulatory hub,” is closely related to the progression of tumors (Figure 2).

Lactate, as a significant intermediary product in both exercise and tumor microenvironments, has a complex biological association with tumor progression. Targeting lactate metabolism and lactylation modifications may provide new directions for cancer therapy. Therefore, this review suggests that regular aerobic exercise can not only influence the tumor environment through the induction of myokines such as irisin and Oncostatin M (OSM) but also reduce lactate levels within the tumor, thereby affecting lactylation modifications. Subsequently, this can further suppress tumor progression by inhibiting the JAK/STAT signaling pathway and other related pathways, restoring the tumoricidal capabilities of immune cells, including CD8^+^ T cells, and regulating the tumor microenvironment.

## Conclusion and prospect

Due to its profound detrimental effects on bodily functions, cancer has become one of the most costly, deadly, and burdensome diseases of this century (Cordani et al., 2024). Simultaneously, the intractability of tumors and their complex prognostic issues pose significant challenges to both society and healthcare systems. It is projected that within the next 50 years, the incidence and mortality rates of cancer will continue to rise, with an anticipated increase of up to 400% in low-income countries. The essential reason for the difficulty in curing tumors lies in the incomplete understanding of their specific pathogenesis and the complexity of associated complications, which generally results in relatively poor prognostic outcomes. Therefore, a thorough analysis of each critical driving factor in tumor development facilitates a better understanding of the mechanisms of cancer onset. Here, this review elucidates the important role of lactate, as a key product of exercise and glycolysis, in tumor growth, as well as the mechanism by which lactate regulates immune suppression through the activation of a novel post-translational modification—lactylation, affecting gene expression and cell signaling pathways, and improving the tumor microenvironment. In the tumor microenvironment, increased activity of MCT and LDH leads to the excessive accumulation of lactate, which, by inducing lactylation at different sites, enhances the activity of oncogenic pathways such as JAK-STAT and PI3K/Akt/mTOR, thereby accelerating tumor cell invasion. Therefore, targeting MCT and LDH as “anti-cancer agents” in immunotherapy offers a new direction for intervening in tumor progression. Currently, clinical inhibitors targeting LDH and MCT mainly include AT-101, NHI-Glc-2, CHC, and BAY8002, which interfere with lactate metabolism and subsequently modulate the lactylation proces (Chen J. et al., 2025). However, due to the complexity of the tumor microenvironment and the involvement of multiple-target regulation in the lactylation process, single-target inhibition still has limitations in clinical treatment outcomes. Aerobic exercise, on the other hand, can regulate the tumor environment by secreting muscle factors such as irisin and myostatin, and can also improve metabolic capacity by modulating the activity of lactate metabolic enzymes, including LDH. This enhances lactate clearance and intervenes in the lactylation modification, restoring the ability of immune cells like CD8^+^ T cells and NK cells to kill tumors. Furthermore, it can improve the body’s immune surveillance against tumors by regulating tumor-related signaling pathways such as JAK-STAT, PI3K/Akt/mTOR, thereby slowing down tumor progression. Moreover, several studies have shown that aerobic exercise can improve the prognosis of various cancers, including breast cancer, lung cancer, and colorectal cance (Kruk et al., 2025). Clinical studies have shown that exercise not only significantly reduces adverse conditions related to tumor prognosis, such as cardiotoxicity, but also improves the body composition of cancer patients, including insulin-like growth factor-1. More importantly, regular exercise can enhance patients’ mental health, physical function, and overall quality of life (Bai et al., 2025; Chen Z. et al., 2025). Additionally, crotonylation, a protein post-translational modification similar to lactylation, also draws attention. Crotonylation involves the enzymatic attachment of crotonyl groups to the ε-amino group of lysine. Although discovered earlier than lactylation, the specific impacts of crotonylation on tumor development, due to similar “writer” and “eraser” enzymes, are still worth exploring in depth. Both modifications, as metabolic and immune bridges, play dual roles in tumor progression. Aerobic exercise, by multidimensionally regulating lactate metabolism and related signaling pathways, offers new opportunities to reshape the tumor microenvironment (TME) and enhance anti-tumor immunity. While the specific mechanisms through which exercise-induced lactylation improves the TME remain unclear, the significant amelioration of the tumor inflammatory environment and the close link between exercise and lactylation may suggest a new potential mechanism for intervening in tumor progression. As deeper molecular mechanisms are uncovered, integrating metabolic regulation, immune activation, and epigenetic measures may unveil new targets for solid tumor therapy, paving a more effective path for treatment and improving cancer prognosis.
